# Development of Au NPs-decorated filter paper as a SERS platform for the detection of benzidine

**DOI:** 10.1039/d1ra05706e

**Published:** 2021-12-14

**Authors:** Rong Wang, Hongyan cao

**Affiliations:** College of Chemical Engineering, Sichuan University of Science and Engineering Zigong 643000 China

## Abstract

In this paper, a simple and cost-efficient strategy was used to construct a uniform Au NPs distribution on the surface of flexible filter paper for the detection of benzidine. Taking full advantage of the adsorption properties of filter paper, small gold nanoparticles were adsorbed onto its surface as gold seeds, and subsequently grown by electroless plating to form a highly uniform distribution of Au NPs substrates. By changing the electroless plating time, an optimal substrate was obtained. The as-prepared substrate exhibited satisfactory sensitivity with a low detection limit of 10^−13^ M for 4-ATP, and good reproducibility and homogeneity. Furthermore, the as-prepared substrates were successfully used for the detection of benzidine in environmental water, with a minimum detection concentration as low as 0.1 ppm and recoveries in the range of 92.4 to ∼108.5%. This study indicated that filter paper-based SERS substrates have great potential value in the detection of environmental organic pollutants.

## Abstract 2

A simple and cost-efficient strategy was used to construct a uniform Au NPs decorated filter paper SERS substrates for the detection of benzidine.

## Graphical abstract

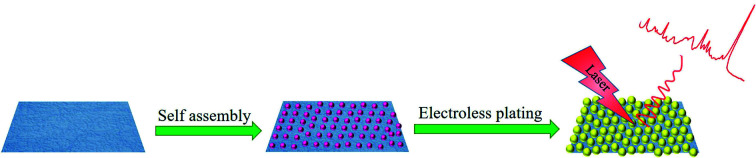

## 1. Introduction

Benzidine is an important industrial raw material with high toxicity, which is mainly used in the production of dyes.^1^ It could cause environmental pollution and affect human health through the discharge of industrial waste water.^2^ Because of its strong carcinogenicity, benzidine has been identified as a class I carcinogen by the International Institute for Cancer Research (IARC).^3^ Therefore, detection of benzidine in the environment is very important. At present, the conventional analytical methods, including high performance liquid chromatography (HPLC)^4,5^ and liquid chromatography tandem mass spectrometry (HPLC-MS),^6,7^ have been used for the detection of benzidine. These methods have high detection sensitivity, but the sample pretreatment process is cumbersome and time-consuming. Moreover, due to the complexity of the instrument, only skilled personnel can use and maintain the equipment, so the cost of testing is higher. Therefore, it is necessary to establish a simple and low-cost method for the determination of benzidine.

Since its discovery in 1970s, surface enhanced Raman spectroscopy (SERS) has been widely used in chemistry, food, environmental monitoring and other fields due to its ability to provide abundant molecular information and high sensitivity.^8–11^ As we all know, the key to the application of SERS technology is to obtain high-performance SERS substrate with high sensitivity, uniformity and reproducibility.^12^ However, up to now, SERS substrate preparation technology is still limited by complex preparation process, uneven distribution of nanoparticles, high preparation cost, poor reproducibility and difficulty in batch preparation.^13–16^ How to simplify the preparation process and obtain excellent SERS active substrate has become an urgent problem to be solved.

In recent years, filter paper has become an ideal material for SERS substrate preparation due to their abundant natural resources, biodegradable, flexibility, low cost,^17^ porous nature, easy cutting^18^ and sample collection.^19–23^ To integrate nanoparticles on flexible paper, various fabrication methods were developed and implemented including soaking, inkjet-printing,^24^*in situ* growth,^22,25^ physical vapor deposition,^26^ filtration. Among these methods, inkjet-printing, filtration and *in situ* growth methods could easily and quickly load nanoparticles on the surface of filter paper, but it does not necessarily result in uniform and orderly distribution SERS substrate.^7,27^ Garnier's group prepared paper-based 3D SERS substrate by soaking, Au NPs were uniformly adsorbed on paper by van der Waals binding without any retention aid,^28^ but this method was difficult to adjust the shape and size of nanoparticles.^29^ Moreover, the self-assembly of nanoparticles on the paper surface in soaking will be affected by the charge repulsion force between nanoparticles, resulting in large particle spacing, so it is difficult to form effective “hot spots”.^1,30–33^

In this work, we developed a self-assembly and electroless plating composite approach for controllable loading of Au NPs on filter paper. This fabrication strategy can easily realize the mass production of filter paper-based Au NPs SERS-active substrate. Most importantly, the filter paper has a 3D porous structure, which was beneficial to load more gold nanoparticles and adsorb more analytes, thus generating stronger SERS sensitivity. Due to the exceptional performance of our prepared Au NPs-decorated filter paper, it was subsequently utilized to detect the concentration of benzidine in environmental water.

## 2. Experimental

### 2.1 Materials and reagents

Chloroauric acid (HAuCl_4_·3H_2_O) and 4-aminothiophenol (4-ATP) were obtained from Aladdin (China). Filter paper (30–50 μm pores) was purchased from Fushun Min zheng Filter Paper Factor. Benzidine, alcohol, anhydrous sodium citrate and hydroxylamine hydrochloride were bought from Chongqing Chemical Reagent Co. Ltd. All chemical reagents were used without further purification. Ultrapure water supplied from a Milli-Q system was used in all experiments.

### 2.2 Synthesis of gold colloids

The Au NPs colloids were prepared according to a previously reported method with minor modificationed.^30^ First, 100 mL of 0.01% (w/w) chloroauric acids solution was heated to boiling with magnetic stirring, then 2 mL of 3% (w/w) sodium citrate was rapidly added, and the solution was kept boiling and magnetic stirring for 15 min. After that, the gold colloid solution was cooled at room temperature. Thereafter, it was kept at 4 °C until use.

### 2.3 Fabrication of Au NPs on filter paper

The filter paper was cut into 1 cm × 1 cm of small pieces of paper and then dipped into the gold colloid for 36 h to form an self assembled monolayer of Au seed. Following this step, the Au seed coated filter papers were thoroughly cleaned with ultrapure water to remove the loosely bound Au NPs. Subsequently, the Au seeds coated filter papers were soaked in the gold plating solution of 0.1% (w/w) chloroauric acids and 0.2 M hydroxylamine hydrochloride to make the gold seeds grow up. After a specified electroless plating time, the substrates were immediately removed from the reactor and repeatedly washed with ultrapure water. The Au NPs decorated filter paper was finally dried at 45 °C.

### 2.4 Characterization

The morphologies of the obtained filter paper based Au NPs substrates were observed through a field-emission scanning electron microscope (Hitachi S4800) at an accelerating voltage of 10 kV. The X-ray diffraction (XRD) patterns were obtained on a X-ray diffractometer (Bruker, D_2_ PHASER) equipped with Cu Kα radiation (*λ* = 1.5406 Å). X-ray photoelectron spectroscopy (XPS) analysis with Al Kα X-ray beams as the excitation source was carried out on an ESCALAB Xi instrument (Thermo Scientific). UV-vis spectra was measured by using UV-visible/NIR spectrophotometer (Hitachi UH4150) with an integrating sphere in the range of 360–900 nm.

### 2.5 SERS measurements

In the SERS experiments, the as-prepared SERS substrates were cut into 2 mm × 4 mm and then immersed into 1 mL 4-ATP solutions with different concentrations for 1 h and dried in air for SERS detection. All normal Raman and SERS spectra were performed on a DXR Microscopes Raman microscope (Thermo Scientific), which equipped with 780 nm diode laser excitation, a 10× objective lens, and a CCD detector. The excitation power was 8 mW and the integration time was 5 s.

#### 2.5.1 Practical application of the proposed SERS platform

For the detection of benzidine, the as-prepared SERS substrates were cut into 2 mm × 4 mm, and then dipped in 1 mL aliquots of different concentrations of benzidine solution ranging from 0.1 ppm to 200 ppm for 1 h and dried in air. Environment water samples were collected from the lake (Zigong, China) to verify the feasibility of the developed method. The representative environment water was filtered through 0.45 μm microfiltration membrane, and spiked with benzidine to the final concentration of 2, 8, or 12 ppm. The Raman test conditions of benzidine were the same as that of 4-ATP detections mentioned above.

## 3. Results and discussion

### 3.1 Preparation and characterization of Au NPs SERS substrates

**Fig. 1:**
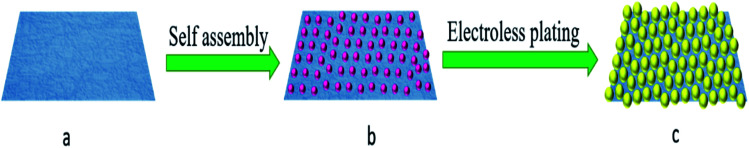
Schematic illustration of the fabrication strategy of Au NPs-decorated filter paper substrate.

**Fig. 2:**
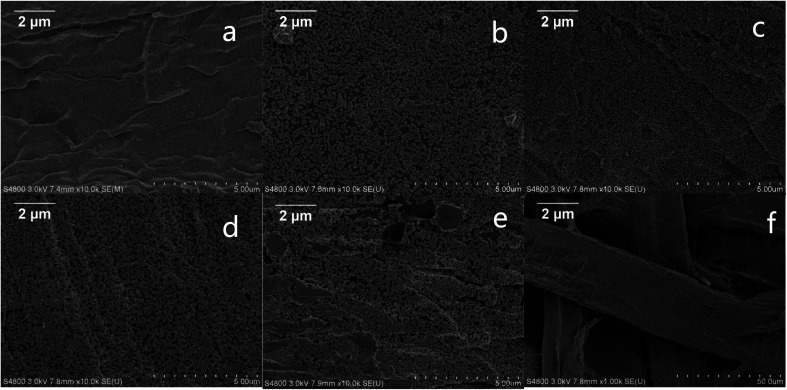
The representative SEM images of the substrates obtained under different chemical plating time solution for 0 min (a), 5 min (b), 20 min (c), 40 min (d), and 60 min (e) respectively; (f) the low magnification SEM image.

In an endeavor to overcome the shortcomings of low sensitivity and poor reproducibility of conventional SERS sensors, we introduced convenient, effective and controllable self-assembly and electroless plating composite method to prepare high-density and uniformly distributed Au NPs on filter paper. The fabrication process is illustrated in Fig. 1. Firstly, gold colloids with Au NPs size of about 22 nm was prepared by the reduction reaction of auric chloride and sodium citrate. Then, filter papers were dipped into the gold colloid. The gold nanoparticles were uniformly adsorbed on the surface of the filter paper fibers by van der Waals binding without any retention aid,^28^ and used as gold seeds. Finally, the filter papers with the gold seed were immersed in the gold-plated solution to grow the gold seeds to the proper size. The surface morphologies of the Au NPs-filter paper with different electroless plating time were characterized by SEM. As shown in Fig. 2a, filter paper was uniformly covered by spheroidal Au seeds with an average diameter of approximately 22 nm, and without any signs of large-scale aggregation following the self-assembly process. After being immersed in a growth solution, the size and coverage of the Au seeds drastically increased and the distances between the Au NPs decreased as the deposition time was increased from 5 to 40 minutes, as shown in Fig. 2b–d. By prolonging the electroless plating time, the particles tended to grow together, which lead to the formation of an Au film on the filter paper (Fig. 2e). It can be seen that the morphology of the products can be easily controlled by adjusting the electroless plating time. As can be seen from the SEM at low magnification (Fig. 2f), the as-prepared substrate has a three-dimensional porous structure.

**Fig. 3:**
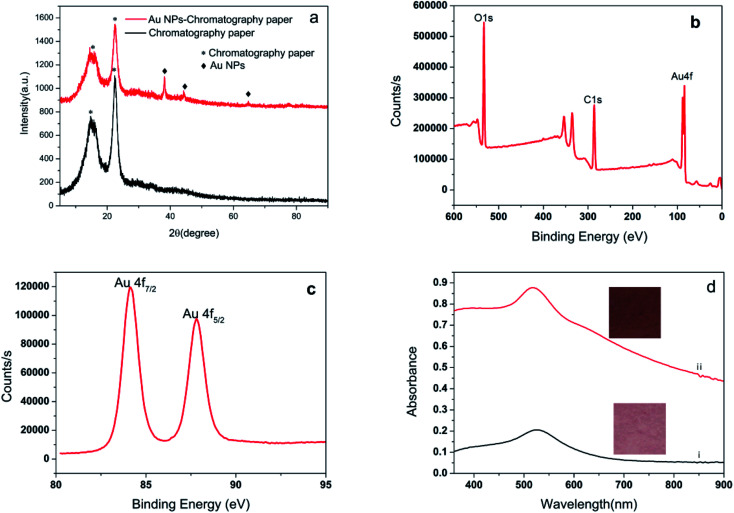
(a) Typical XRD pattern of filter paper and Au NPs-decorated filter paper; (b) XPS wide-scan spectra of Au NPs-decorated filter paper; (c) XPS spectra of Au 4f; (d) UV-vis spectra of paper Au NPs-decorated filter before (i) and after electroless plating (ii) and respective digital photographs.

The compositions of the as-prepared SERS substrate was characterized by using XRD, and a representative pattern is depicted in Fig. 3a. The broad peak at around 14.7 and 22.5° (marked with *) originates from the filter paper.^34^ The diffraction peaks with 2*θ* values of 38.1°, 44.3°, 64.4°, and 77.5° can be assigned to the 111, 200, 220, and 311 crystallographic planes of Au, respectively.^35–37^ This confirms that the filter paper was coated with crystallized Au (JCPDS no. 04-0784).^38^ The XPS pattern in Fig. 3b and c represents the signature of the Au 4f doublet (4f_7/2_ and 4f_5/2_). The Au 4f_7/2_ and Au 4f_5/2_ peaks at 84.1 and 87.8 eV are consistent with the Au_0_ state.^39^ The appearance of the Au_4f_ peaks further confirming that gold is integrated into the filter paper. We record the UV-vis spectra of the Au NPs-filter paper (Fig. 3d) before and after electroless plating, and found than the plasmon peak increased and broadened significantly after 40 min of electroless plating, which was ascribed to strong interparticle plasmonic coupling caused by the gap decreases with the increase of Au NPs size, which is more conducive to enhancing SERS signal.

**Fig. 4:**
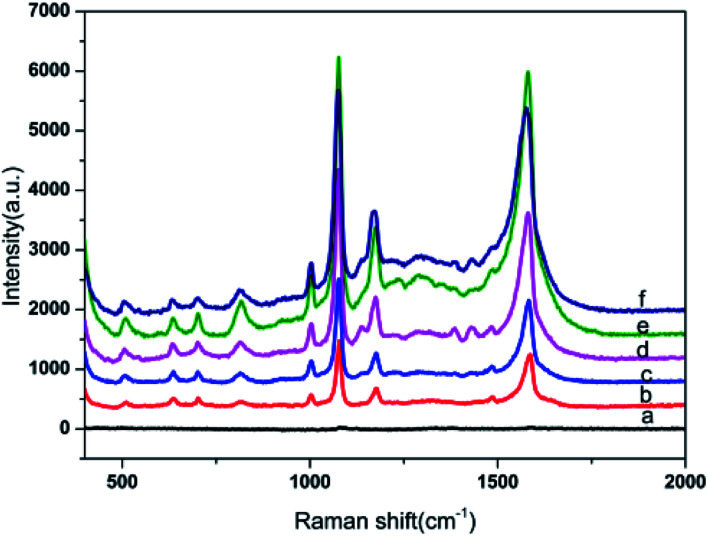
SERS spectra of 10^−6^ M 4-ATP using Au NPs-decorated filter paper substrate obtained at different electroless plating time: 0 min (a), 5 min (b), 10 min (c), 20 min (d), 40 min (e),and 60 min (f).

The 4-ATP was used as the probe molecule, and the SERS performance of the prepared substrate under different electroless plating times were investigated. The results are shown in Fig. 4. All spectra clearly indicate that there were no significant differences except in the intensity of the SERS signal. The prominent peaks centered at 1003, 1078, 1178 and 1581 cm^−1^, were consistent with the Raman spectra of 4-ATP molecules reported in the literature.^40,41^ The SERS signal intensity of the substrates prepared without electroless plating was the lowest, because only sparse Au NPs were adsorbed onto the surface of the filter paper. It was ultimately noted that the intensity of the SERS signal for 4-ATP gradually increased along with the increase in the electroless plating time, and reached a maximum at 40 minutes and then declined with prolonging of the electroless plating time. It can therefore be argued that this was a reasonable outcome, because the Au nanoparticles became larger in relation to the increase in electroless plating time and the interspacing between the Au NPs decreased. This resulted in the boosting of the SERS signals due to the enhanced electromagnetic coupling between the neighboring nanoparticles. This could lead to improved SERS performance as a result of a higher EM. Should the deposition time however be longer than 40 min, some neighboring Au NPs merge together and grow in size, the gaps gradually disappear and a thin Au film forms (Fig. 2e), which causes a decrease of potential ‘hot spots’.

### 3.2 Evaluating the sensitivity of paper based Au NPs SERS substrates

**Fig. 5:**
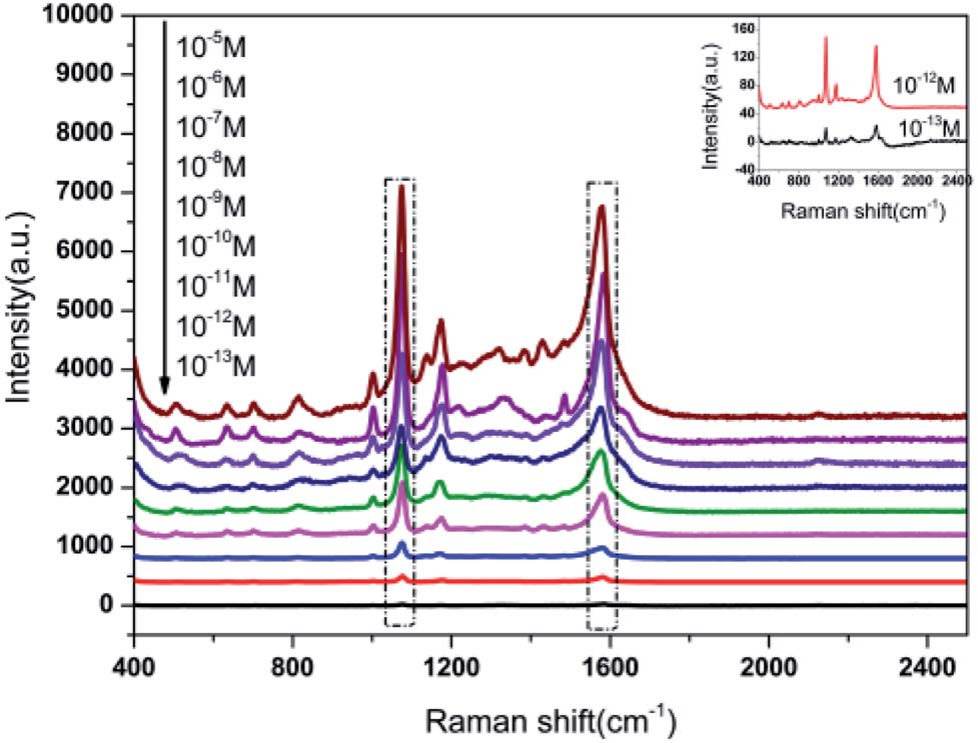
SERS spectra of 4-ATP with different concentrations.

To evaluate the sensitivity of Au NPs-decorated filter paper substrate, we measured the 4-ATP spectra for a wide range of molar concentrations ranging from 10^−6^ to 10^−13^ M, and their SERS spectra are shown in Fig. 5. From the plot, it is clear that the main SERS band at 1003, 1078, 1178 and 1581 cm^−1^ were easily observed for 10^−11^ M of 4-ATP. Moreover, the characteristic peaks of 4-ATP were visible up to 10^−13^ M in an enlarged SERS image which was lower than other paper-based SERS substrate previously reported by other research groups.^28^ The results revealed that as-prepared SERS substrate had higher sensitivity for SERS detection.

### 3.3 SERS reproducibility test

**Fig. 6:**
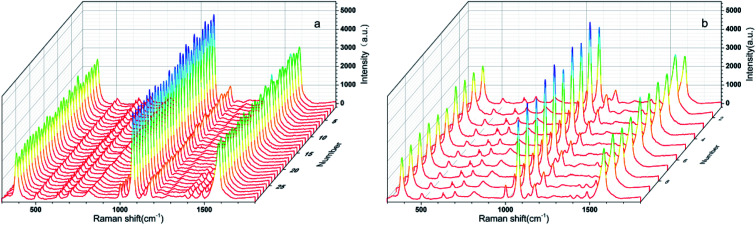
(a) SERS spectra of 4-ATP randomly recorded at 30 selected spots. (b) SERS spectra of 4-ATP randomly recorded at ten different Au NPs-decorated filter paper substrate.

As we all know, a good SERS substrate exhibits not only high enhancement ability but also good reproducibility across both a single as well as multiple substrates in practical application. To investigate the uniformity of the Au NPs-decorated filter paper substrate, the SERS spectra of 10^−6^ M 4-ATP was acquired at 30 randomly selected positions on the same substrate, and the data were depicted in Fig. 6a. The relative standard deviation (RSD) of SERS intensity at the main peaks of 1003, 1078, and 1581 cm^−1^ were 7.8%, 4.7%, 5.7%, respectively, indicating good signal uniformity, which can be attributed to the uniform distribution of Au NPs on the paper surface. To further evaluate the repeatability of the preparation process, the signal variations from 10 different Au NPs decorated filter paper substrates were also investigated, as shown in Fig. 6b. The RSD value of SERS intensity was 9.1%, 7.2% and 8.3% corresponding with the band at 1003, 1078, and 1581, respectively. The results fully showed that the SERS substrate prepared by the method established in this paper had good uniformity and reproducibility, and the preparation process of SERS substrate was simple and controllable, which was very suitable for batch production.

### 3.4 Application for detection of benzidine in environmental water

**Fig. 7:**
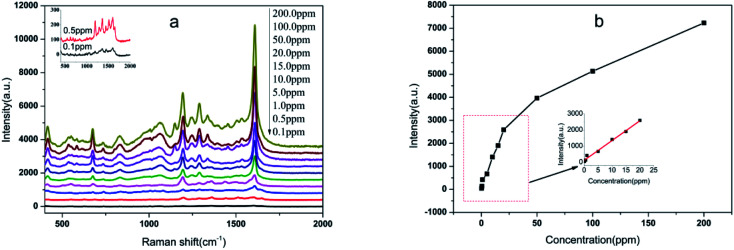
(a) Typical SERS spectra of gradient concentrations of benzidine; (b) function of the peak intensity at 1607 cm^−1^ of benzidine with concentration. Inset: a linear fitting for peak intensity at 1607 cm^−1^ with the concentration ranging from 0.1 ppm to 20 ppm.

To further investigate the practical application of the Au NPs-decorated filter paper substrate, a test of organic pollutants relevant to environmental monitoring was performed. Benzidine is a typical dye precursor in the pigment industry. Unfortunately, the wide use of this chemical has led to environmental pollution and a threat to human health. The SERS spectra of benzidine with concentrations from 0.1 ppm to 200 ppm were obtained as shown in Fig. 7a. The characteristic peaks at 847, 1194, 1282 and 1610 cm^−1^ of benzidine can be clearly observed even at a very low concentration of 0.1 ppm. In the range of 0.1–20 ppm, there was a good linear relationship (*R*^2^ = 0.989) between the peak intensity at 1607 cm^−1^ and benzidine concentrations, with a linear equation of *I* = 123.32 + 122.16*C* (ppm) (Fig. 7b). Furthermore, in the spike-and-recovery experiment carried out on environment water, the recoveries are in the range of 92.4–108.5%, with RSD values of 5.6–8.1% (Table 1). This means that the substrate in the present study has the potential for analysis of trace benzidine in environment water.

**Table 1:** Detection values of benzidine based on the spike-and-recovery experiments carried out environmental water samples (*n* = 5)^a^

Spiked concentrations (ppm)	Found concentrations (ppm)	Recovery (%)	RSD (%)
2.0	2.17	108.5	8.1
8.0	7.39	92.4	6.4
12.0	12.45	103.8	5.6

a Recovery = (found concentration/spiked concentration) × 100%.

## 4. Conclusions

In summary, a filter paper based Au NPs SERS substrate was successfully prepared by a simple self-assembly and electroless plating composite method and applied to the rapid detection of benzidine in environmental water samples. The synthesized substrates exhibit excellent SERS performance with good enhancement ability and reproducibility between single and multiple substrates. Furthermore, the presented SERS substrate was proved capable of detecting benzidine in environment water at the 0.1 ppm level. Overall, the developed Au NPs-decorated filter paper substrate has been proven to be an efficient SERS-active substrate for the detection of benzidine, which provided a promising solution for the detection of organic pollutants in the environment by SERS technology.

## Conflicts of interest

There are no conflicts to declare.

## Supplementary Material
